# Editorial: Beyond borders: exploring diverse roles of heterocyclic compounds in combatting infections and cancer

**DOI:** 10.3389/fchem.2025.1578852

**Published:** 2025-03-04

**Authors:** Afzal B. Shaik, Richie R. Bhandare, M. Mukhlesur Rahman, Kamal Dua

**Affiliations:** ^1^ Department of Pharmaceutical Sciences, School of Biotechnology and Pharmaceutical Sciences, Vignan’s Foundation for Science, Technology and Research, Guntur, India; ^2^ Center for Global Health Research, Saveetha Medical College, Saveetha Institute of Medical and Technical Sciences, Chennai, Tamil Nadu, India; ^3^ College of Pharmacy & Health Sciences, Ajman University, Ajman, United Arab Emirates; ^4^ Center of Medical and Bio-Allied Health Sciences Research, Ajman University, Ajman, United Arab Emirates; ^5^ The Medicines Research Group, School of Health, Sports and Bioscience, University of East London, London, United Kingdom; ^6^ Faculty of Health, Australian Research Centre in Complementary and Integrative Medicine, University of Technology Sydney, Ultimo, NSW, Australia; ^7^ Discipline of Pharmacy, Graduate School of Health, University of Technology Sydney, Ultimo, NSW, Australia

**Keywords:** bioactive heterocyclic compounds, medicinal chemistry, anti-infective agents, anticancer agents, natural product chemistry, computational chemistry, synthesis and semisynthesis, drug design

This Research Topic aimed to explore the advancements and applications of heterocyclic compounds, highlighting their importance in contemporary medicinal chemistry. The objective was to examine their diverse roles in tackling two major health concerns: infectious diseases and cancer. Currently, cancer and infectious diseases are among the most prevalent and challenging health conditions, significantly affecting the overall wellbeing of the population. According to the World Health Organization (WHO), approximately 20 million new cancer cases were diagnosed globally in 2022, with 9.7 million deaths attributed to the disease. Projections suggest that by 2050, the number of cancer cases could rise to 35 million (Seigel et al., 2023). Antimicrobial resistance (AMR) is recognized as one of the top ten global health threats, posing significant risks not only to human health but also to environmental wellbeing. It is classified as a quintessential One Health challenge. The most concerning pathogens, characterized by multidrug resistance (MDR), extended-drug resistance, and even pan-drug resistance phenotypes, are collectively referred to by the acronym ESCAPE, encompassing *Enterococcus faecium*, *Staphylococcus aureus*, *Clostridium difficile*, *Acinetobacter baumannii*, *Pseudomonas aeruginosa*, and *Enterobacteriaceae* (De Oliveira et al., 2020). Recent developments in this area have shown the potential of heterocyclic compounds to offer more targeted and effective treatments with fewer side effects. Therefore, this Research Topic invited papers that showcased recent progress in medicinal and natural product chemistry, including the isolation and characterization, design, synthesis, and application of heterocyclic compounds as promising anti-infective and anticancer agents.

This Research Topic covers a total of fourteen original research articles investigating ligands spanning broad therapeutic areas like breast/prostate cancer, inflammatory bowel disease, antitubercular ligands, antimicrobials, anticholinergic and antidiabetic. Seven papers focused on exploring heterocyclic compounds from natural products using various techniques such as molecular dynamics, nanoparticles, *in silico* and GC-MS profiling and quantum analysis while the remaining papers emphasised the use of heterocyclic fragments for various therapeutics activities.

Mashud et al. investigated the potential inhibitory effects of specific compounds present in leaf extract from *Mangifera indica* on the growth of drug-resistant breast cancer protease. The chemical compounds present in the plant were analysed using molecular modelling techniques, such as molecular docking, molecular dynamics (MD) simulations, quantum mechanics (QM) calculations, and the Absorption, Distribution, Metabolism, Excretion, and Toxicity (ADMET) method, in order to examine three key chemical constituents: quercetin, catechin, and ellagic acid. The ligands underwent extensive testing to determine their effectiveness against the 3w32-overexpressing breast cancer protein. This study used molecular docking tools and molecular dynamic studies identified ligands with strong binding affinity for the breast cancer protein that overexpressed 3w32. The study identified three ligands that not only surpassed the efficacy of the FDA-approved treatment, but also fulfilled the requirements for a possible new inhibitor of breast cancer.

Barik et al. explored the potential use of silver nanoparticles (AgNPs) for treating periodontal infections, synthesized using leaf extract from *Azadirachta indica*. The eco-friendly green synthesis process utilizes the plant as a natural stabilizer and reducer, facilitating the formation of silver nanoparticles. Various analytical techniques, including transmission electron microscopy (TEM), Fourier-transform infrared spectroscopy (FTIR), Zeta potential analysis, and ultraviolet-visible spectroscopy (UV-Vis), were employed to characterize the AgNPs. The antimicrobial and antioxidant properties of AgNPs were tested to evaluate their effectiveness against periodontal infections. The results demonstrate significant antibacterial and antioxidant activity, inhibiting biofilm formation and bacterial viability. The study has suggested that AgNPs derived from *A. indica* may serve as a safe, effective, and environmentally friendly alternative to traditional therapies for treating periodontal infections.

Mallela et al. investigated the anticancer effects of methanolic extracts from Lotus seeds (MELS) on cell proliferation inhibition, apoptosis induction, and cell cycle arrest in ovarian cancer cell lines. They also reported the phytochemical composition of MELS using gas chromatography-mass spectrometry (GC-MS) analysis. Additionally, molecular docking studies were conducted to support the *in vitro* anticancer effects by examining the inhibitory potential of MELS on human survivin protein. The *in-vitro* findings demonstrated significant inhibition of SKOV3, A2780, SKOV3-CisR, and A2780-CisR cells by MELS, in comparison to acetone, petroleum ether, n-hexane extracts, and the standard drug, cisplatin. They employed GC-MS to analyse and characterize 14 potential phytocompounds present in MELS. Molecular docking results showed that oleic acid, stigmast-5-en-3-ol, phytol, and glyceryl linolenate exhibited strong binding affinities to survivin. These findings suggest that the phytochemicals identified in MELS may have therapeutic potential for the management of ovarian cancer.

Kirboga et al. reported the binding affinities and interaction profiles of selected cannabinoids and stilbenoids on eight proteins through molecular docking and molecular dynamics simulations. They identified ligands with the highest binding affinities, and their pharmacokinetic properties were assessed using ADMET analysis. The results revealed that GMP synthase showed the strongest binding affinity with Cannabistilbene I, indicating hydrophobic interactions and multiple hydrogen bonds. Similarly, Chitin Synthase 2 also demonstrated significant binding with Cannabistilbene I. In contrast, ligands like Cannabinolic acid and 8-hydroxycannabinolic acid exhibited moderate binding affinities, highlighting the variability in interaction strengths across different proteins. Experimental validation is essential to confirm their therapeutic potential. This study is pivotal for future research, emphasizing the importance of evaluating binding affinities, pharmacokinetic profiles, and multi-target interactions to identify promising antifungal agents.

Belal et al. focused on the antiviral properties of natural indoles, Gardflorine A–C, extracted from *Gardneria multiflora* Makino. Utilizing molecular docking, ADMET analysis, and computational approaches—including Frontier molecular orbital (FMO) analysis, natural bond orbital (NBO) analysis, and density functional theory (DFT)—the authors evaluate these compounds’ potential as multi-target antiviral ligands against HIV and HCV proteins.

Edis et al. highlighted the potential of clove extract-mediated nanoparticle synthesis as an effective approach for integrating medications with metals at the nanoscale. These nanoparticles exhibited a synergistic effect with the heterocyclic antibiotic clarithromycin, enhancing its therapeutic efficacy, reducing side effects, and improving antimicrobial activity. The authors synthesized silver nanoparticles using clove extract, resulting in AgNPC and AgNPCA (A = clarithromycin). Various instrumental techniques, including SEM, EDS, DLS, UV-Vis, FTIR, and XRD, were used to analyze the compounds. When tested against different microbes, the nanoparticles demonstrated antibacterial properties ranging from intermediate to strong. The study underscores the potential of clove extract-mediated AgNP synthesis, both alone and in combination with clarithromycin.

Altin et al. described the therapeutic potential of *Laurus nobilis* leaves, emphasizing their rich phenolic content and bioactive properties, including antioxidant, antidiabetic, and anticholinergic effects. The phenolic compounds in the ethanolic extracts were analysed using LC-MS/MS, while antioxidant activity was assessed through ferric thiocyanate, DPPH, ABTS assays, and metal reduction potential tests. Anticholinergic and antidiabetic properties were evaluated via inhibition studies on acetylcholinesterase (AChE), butyrylcholinesterase (BChE), and α-glucosidase (α-GLY) enzymes, complemented by *in silico* analysis to investigate binding mechanisms. Vanillic acid and catechin hydrate were identified as the most abundant phenolics, with the extract demonstrating superior lipid peroxidation inhibition compared to Trolox and α-tocopherol. Moderate radical scavenging activity and metal reduction potential further supported its bioactivity. In silico results revealed a strong binding affinity of phenolics to target enzymes, reinforcing their therapeutic potential. This study has highlighted the promising antioxidant, antidiabetic, and anticholinergic properties of *L. nobilis* extracts for potential medicinal applications.

Thakur et al. designed novel analogues of Cabozantinib (CBZ) using a bioisosteric approach to develop compounds with reduced toxicity, improved safety, and enhanced potency against multi-drug-resistant cancers. The physicochemical, medicinal, and ADMET properties were assessed using ADMETLab 3.0. Additionally, the authors evaluated the drug-likeness and drug scores of the analogues. Molecular docking studies were conducted using AutoDock Vina, with BIOVIA Discovery Studio employed for visualizing key interactions. The docking scores for the ligands ranged from −8.0 to −6.4 kcal/mol against the target protein. Molecular dynamics (MD) simulations of selected compounds, performed using the Schrödinger suite, demonstrated that the complexes remained stable throughout the simulation period. This study has highlighted the potential of two promising ligands as candidates for the development of novel anticancer agents for treating various cancers.

Al-Wahaibi et al. described total synthesis of a series of disalicylic acid methylene/Schiff base hybrids designed to function as antibacterial agents by targeting DNA gyrase and DHFR. Their study identified several compounds with potent antibacterial activity against both Gram-positive and Gram-negative bacteria, demonstrating inhibition zones (IZ) comparable to or exceeding those of the reference drug Ciprofloxacin.

Shahi et al. focused on developing dihydropyrimidinone and dihydropyridine derivatives of thymol and evaluating their antimicrobial properties. The synthesized compounds showed broad spectrum *in-vitro* antibacterial activity against *P. aeruginosa* and methicillin-resistant *S. aureus* (MRSA). Among the derivatives, they identified a promising compound with the most potent antibacterial activity against both *P. aeruginosa* and MRSA. Furthermore, the most potent compound exhibited synergistic effects when combined with vancomycin, enhancing its antibacterial efficacy. In silico analysis of its physicochemical properties confirmed compliance with all drug-likeness criteria. Lastly, molecular docking studies revealed that the promising compound had a stronger binding affinity to the target protein than thymol, providing valuable insights into its potential mechanism of action.

Gupta et al. computationally designed novel analogues of Apalutamide with the aim of enhancing pharmacokinetic properties and minimizing toxicity. Drug-likeness (DL) and drug score (DS) were also assessed. Molecular docking and molecular dynamics (MD) simulations were performed to evaluate the binding affinities of the designed analogues and compare their binding orientations with those of the ligands in the original crystal structure. The findings indicate that two analogues exhibit potential as antiandrogen ligands for the treatment of prostate cancer.

Sabt et al. synthesized a series of novel compounds by conjugating 4-carboxyquinoline with triazole motifs. These compounds were evaluated for their antimicrobial efficacy against various *Mycobacterium* strains, including *M. bovis* BCG, *M. tuberculosis*, and *M. abscessus*. Additionally, their inhibitory potential against the InhA enzyme was assessed. Several molecules demonstrated significant activity against *M. tuberculosis*. Molecular docking analysis revealed key interactions between these compounds and the target enzyme, while molecular dynamics (MD) simulations confirmed the stability of the quinoline-triazole conjugate complexes with InhA. Furthermore, the most potent compound underwent *in silico* ADME analysis to predict its pharmacokinetic properties. This study provides valuable insights into the development of novel, safe, and effective small-molecule therapeutics for tuberculosis treatment.

Arif et al. reported the development of a novel isatin derivative capable of degrading estrogen receptor alpha (ERα) in estrogen-dependent breast cancer cells. A series of hydrazide derivatives were synthesized and evaluated *in vitro* for their antiproliferative activity against the MCF-7 (ER+) cell line. The effect of the most potent compound on ERα expression was further analyzed using Western blot analysis. Additionally, *in silico* pharmacokinetic predictions were conducted using various computational tools, including pkCSM, to assess the activity profiles of the synthesized compounds.

Zhao et al. identified novel chrysin derivatives with promising therapeutic potential for inflammatory bowel disease (IBD). Among them, a potent compound exhibited significant inhibitory activity against TNF-α-induced monocyte adhesion to the colonic epithelium. Mechanistic studies revealed that this compound suppresses reactive oxygen species (ROS) production and downregulates the expression of ICAM-1 and MCP-1, key mediators of monocyte-epithelial adhesion, as well as the transcriptional activity of NF-κB. *In vivo* experiments further demonstrated its efficacy in mitigating colitis in animal models. These findings highlight the compound’s potential as a promising candidate for IBD management.

In summary, the fourteen original articles featured in this Research Topic have presented recent advancements in the application of heterocyclic scaffolds across various therapeutic areas, including breast cancer, inflammatory bowel disease, and tuberculosis. The collective findings of these studies offer valuable insights for researchers in organic and medicinal chemistry, particularly those focused on chemotherapy. This Research Topic has also emphasized the challenge of antimicrobial resistance and underscores the significance of heterocycles as privileged structures in drug design, making it highly relevant to Frontiers readers in this field. The heterocyclic ring systems discussed herein may be of particular interest to medicinal chemists for the synthesis of bioactive compounds and the development of novel analogues, thereby contributing to chemotherapy drug discovery efforts and addressing the global issue of resistance.
